# The preliminary development and clinical verification of the positive index score scale of “Heart Arthralgia Syndrome”

**DOI:** 10.1097/MD.0000000000034644

**Published:** 2023-09-15

**Authors:** Sai Xu, Shouqiang Chen, Yunsheng Xu

**Affiliations:** a Shandong University of Traditional Chinese Medicine, Jinan, China; b Second Affiliated Hospital of Shandong University of TCM, Jinan, China.

**Keywords:** clinical verification, heart arthralgia syndrome, pick complex therapy, positive index score scale of “Heart Arthralgia Syndrome”

## Abstract

**Background:**

In recent years, the age of onset for coronary heart disease (CHD) has become one of the leading causes of death worldwide. The medical treatments occasionally cause side effects; therefore, there is still an urgent need to develop new therapeutic modalities for CHD in clinical practice. "Heart Arthralgia Syndrome (HAS)" is a general term for CHD with arthralgia symptoms proposed by our team based on clinical experience. At present, there is little in-depth research on the treatment of HAS by TCM. Pick Complex Therapy (PCT) is an innovative and developed theory of collateral acupuncture therapy for HAS.

**Methods:**

We collected data from 276 patients who met the criteria for (coronary heart disease with numbness of neck, shoulder, waist, and leg). We selected 24 diagnostic criteria for HAS by means of multiple methods, including Cronbach’s α coefficient, retest reliability, subjective evaluation, discrete trend, Pearson’s rank correlation coefficient and factor analysis method. We thereafter evaluated the reliability, validity and responsiveness of the scale. In the clinical validation phase, we verified whether the preliminary developed positive index (PI) scale can guide clinical practice. Forty (40) patients with HAS were selected in the study. SPSS23.0 statistical software was used for statistical processing and analysis.

**Results:**

Assessment results of the initial PI scale for HAS: the average time to complete the scale was 7.47 ± 3.59 minutes. Cronbach’s α coefficient for the initial table was 0.711, the retest reliability was 0.897, the Kaiser-Meyer-Olkin test result was 0.844, and the Bartlett test result was 2502.300. Following maximum variance rotation analysis, the cumulative variance contribution rate was determined to be 66.605%. In the clinical validation phase of the PI scale, we tested 40 patients before and after the PCT treatments. After 3 measurements, the correlation between the PI scale for HAS and the angina pectoris grading scoring method table decreased gradually. The last 2 measurement results of study indicated that there was a significant correlation between the PI scale and thrombin time, while physical and chemical examination showed no significant changes.

**Conclusion:**

The PI scale for HAS can be widely used in the clinic as a preliminary evaluation tool for guiding PCT.

## 1. Introduction

Cardiovascular disease refers to systemic vascular disease or manifestations of systemic vascular disease in the heart that leads to an increased risk of heart attack, heart failure, sudden death, stroke, and arrhythmia.^[1]^ In recent years, the age of onset for Cardiovascular disease has decreased, with Cardiovascular disease becoming one of the leading causes of death worldwide. To reduce the risk of Cardiovascular disease, hundreds of thousands of adults annually are treated `with medication, including hypoglycemic drugs, blood pressure medications (such as diuretics and beta-blockers), blood thinners, or antiarrhythmic drugs^[2]^ However, these treatments occasionally cause side effects such as shortness of breath, fatigue, heat shock, headache, and dizziness.^[3]^ Therefore, there is still an urgent need to develop new therapeutic modalities for Cardiovascular disease in clinical practice. “Heart Arthralgia Syndrome (HAS)” is a general term for coronary heart disease (CHD) with arthralgic symptoms proposed by our team based on clinical experience. Unlike the simple arthralgic symptoms in traditional Chinese medicine (TCM), HAS is characterized by CHD with neck, shoulder, back and leg pain and other arthralgia.

Pick complex therapy (PCT) is an innovative and developed theory of collateral acupuncture therapy that combines meridian syndrome differentiation, effective basic substances, Qi street, information conduction, and collateral-disease theories. By cutting off Yang collaterals on the sagittal and coronal surfaces of the body, acupuncture, which extrudes a certain amount of tissue fluid or blood to treat limb meridian diseases, which is often used in the treatment of paralytic and painful diseases and has significant curative effect.^[4]^

At present, HAS is predominantly viewed as 2 separate diseases – CHD and arthralgia syndrome – which are treated with Western medicine and integrated Chinese and Western medicine, respectively. We contend that CHD and arthralgia syndrome are, in fact, related as cause and effect, respectively. There is little in-depth research on the treatment of HAS by TCM. Although PCT is classified as a surgical method, it requires lower oral drug doses than most other surgical treatments and uses both internal and external treatment methods. Therefore, PCT may play a considerable complementary role in the treatment of HAS.

**Figure 1.**
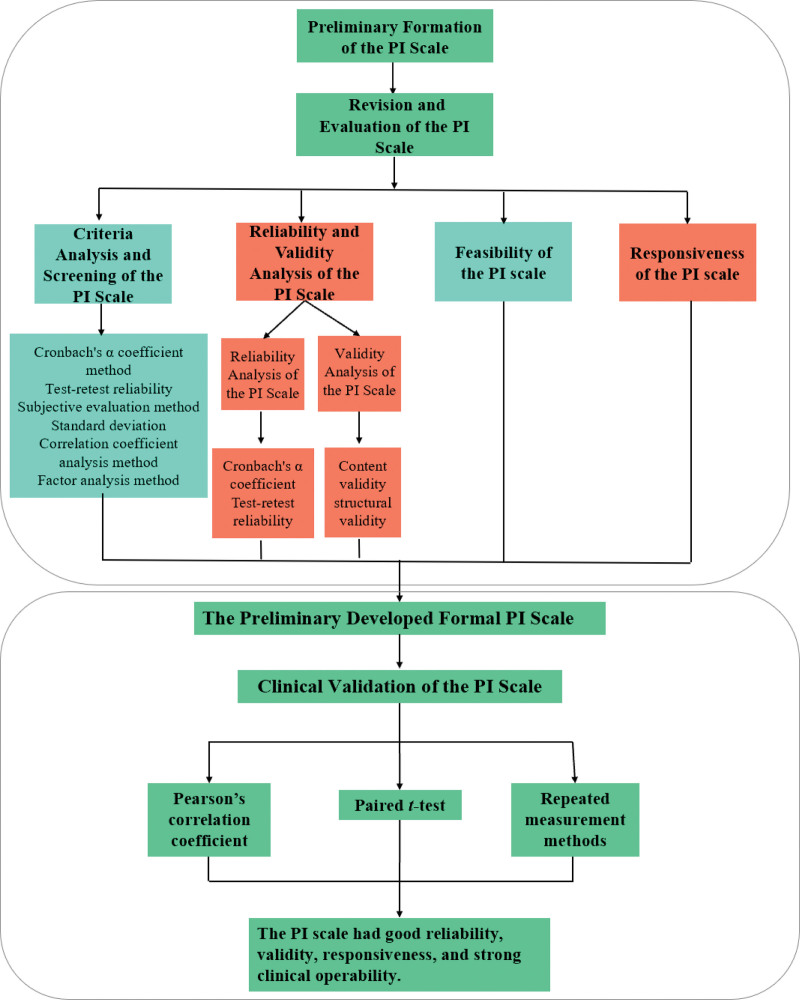
The workflow of this paper. PI = positive index.

The purpose of this study is to complete the preliminary formulation, evaluation, and clinical verification of a positive index (PI) scale for HAS. We expect that this scale may be widely used in clinical practice as a metric for evaluating PCT. The workflow of this paper is illustrated in Figure 1.

## 2. Materials and Methods

### 2.1. Preliminary formation of the PI scale

Based on our team’s preliminary clinical study, we developed a primary scale with 24 criteria, divided into 4 categories, as follows: CHD (chest tightness, chest pain, palpitation); patient self-examination (acidity, numbness, swelling, redness and lump, mild, moderate, and severe pain); doctor’s observation (rash point, spot, stagnated point, choroid point, granular point, and nodular point); doctor’s palpation (gritty shape, nodular shape, ropy shape, lumpy shape, hardness, slight, moderate, and obvious tenderness). A total score of 24 points is possible, with a score of 4 or more indicating a positive candidate pick area (Table 1).

**Table 1:** Initial PI Scale for HAS.

Pick area lookup method	Criterion	Assignment	Selection (if yes, choose √)
Coronary heart disease	Chest tightness	2	
	Chest pain	2	
	Palpitation	2	
Patient self-examination	Acidity	1	
	Numbness	1	
	Swelling	1	
	Redness and Lump	1	
	Mild pain	0.5	
	Moderate pain	1	
	Severe pain	1.5	
Doctor’s observation	Rash point	0.5	
	Spot	0.5	
	Stagnated point	0.5	
	Choroid point	0.5	
	Granular point	0.5	
	Nodular point	0.5	
Doctor’s palpation	Gritty shape	1	
	Nodular shape	1	
	Ropy shape	1	
	Lumpy shape	1	
	Hardness	1	
	Slight tenderness	0.5	
	Moderate tenderness	1	
	Obvious tenderness	1.5	
The total score			

### 2.2. Revision and evaluation of the PI scale

#### 2.2.1. Research subjects.

The majority of our research subjects were HAS patients (i.e., CHD patients with neck, shoulder, waist, leg pain or other arthralgic symptoms). The cases came from a total of 276 patients with “heart paralysis syndrome” collected by the Department of Cardiology within the Second Affiliated Hospital of Shandong University of TCM. All selected patients were diagnosed by Western medicine as exertional angina pectoris of CHD with arthralgia of the neck, shoulder, waist, or leg. All patients were treated with PCT in the outpatient ward of our hospital. This study was conducted with patients’ knowledge and consent and was reviewed and approved by the ethics committee of our hospital.

#### 2.2.2. Diagnostic criteria for CHD using western medicine.

According to the report “Nomenclature and Diagnostic Criteria for Ischemic Heart Disease” from the Joint Task Group on Standardization of Clinical Nomenclature of International Society and Federation of Cardiology and World Health Organization (WHO):

##### 2.2.2.1. Angina pectoris of effort.

Angina pectoris of effort is characterized by a brief attack of chest pain induced by exercise or other circumstances that increase myocardial oxygen demand. After rest or sublingual administration of Nitroglycerin, the pain often disappears quickly. Angina pectoris of effort can be divided into 3 categories:

1. Initial onset angina pectoris: the course of the disease is within 1 month;
2. Stable angina pectoris: The course of the disease is stable for more than 1 month;
3. Progressive angina pectoris: The number, severity, and duration of induced fatigue of the same degree suddenly increase.

##### 2.2.2.2. Grading diagnostic criteria for angina pectoris of effort.

According to the symposium on the treatment of CHD, angina pectoris, and arrhythmia by combining traditional Chinese and Western medicine, “evaluation criteria for CHD, angina pectoris and electrocardiogram efficacy” (Shanghai, September 1979), it is divided into 4 levels:

I: No symptoms of daily activities, heavier physical activities than daily activities cause angina, such as jogging on the ground, fast or holding heavy objects up the third floor, uphill cause angina pectoris;

II: Daily physical activity causes angina pectoris, daily activities are slightly limited, such as walking at normal speed for 3 to 4 stations (3–4 miles) under normal conditions, up the third floor, uphill cause angina pectoris;

III: Physical activities that are lighter than daily activities cause angina pectoris, and daily activities are obviously limited, such as walking at normal speed for 1 to 2 stations (1–2 miles) under normal conditions, up the second floor or small slope cause angina pectoris;

IV: Mild physical activity (such as walking slowly indoors) causes angina pectoris, and in severe cases angina pectoris also occurs when resting.

This study only selected patients who met the grading of stable angina pectoris in grades I–III.

#### 2.2.3. Criteria analysis and screening the PI scale.

Cronbach’s α coefficient, test-retest reliability, subjective evaluation, discrete trend, correlation coefficient, factor analysis, and other methods were used to screen the 24 criteria of the PI scale.

##### 2.2.3.1. Cronbach’s α coefficient method.

Cronbach’s α coefficient for the scale and for each category was calculated to evaluate the internal consistency of the scale. It is generally held that the α coefficient should be above 0.8, but most studies believe that the Cronbach’s α coefficient > 0.6 is credible, indicating good internal consistency among criteria.^[5]^

##### 2.2.3.2. Test-retest reliability.

Patients were asked to fill in the scale again within 12 to 24 hours of receiving PCT. Due to poor patient compliance, we collected data from only 92 patients out of the original 276 patients treated with PCT for HAS. Each of these 92 patients was measured twice, once within 12 to 24 hours before treatment, and once within 12 to 24 hours after. From these measurements, Pearson’s rank correlation coefficient for the 2 scores for each criterion was calculated, and criteria with a correlation coefficient > 0.7 were retained.

##### 2.2.3.3. Subjective evaluation method.

Sixteen (16) clinicians and nursing staff independently evaluated the importance of each criterion proposed by the scale according to their own knowledge and clinical experience. Criteria were graded on 3 levels, namely unimportant, relatively important, or very important, and assigned 0, 50, and 100 points respectively. The average score of each criterion was calculated, and criteria with an average score ≥ 50 points were selected.^[6]^

##### 2.2.3.4. Standard deviation.

Standard deviation (SD) was used to measure the degree of data dispersion. The larger the SD, the more sensitive the item.^[7]^ The index Criteria with the smallest coefficient of variation SD < 0.2 was viewed to have limited differential diagnostic utility, and thus discarded.^[8]^

##### 2.2.3.5. Correlation coefficient analysis method.

This method uses 2 correlation coefficient methods to screen criteria from 2 perspectives^[9]^: the representative correlation coefficient and independent correlation coefficient. The representative correlation coefficient examines the correlation between each criterion and the total score of the initial scale. Since the scores in this scale are discrete data, we analyzed them with Spearman’s rank correlation coefficient. Any criterion with a correlation coefficient < 0.4 was discarded. The independent correlation coefficient shows that 2 or more Spearman’s rank correlation coefficients ≥ 0.4 between the criterion’s own category and scores in other categories, indicating independent correlation is poor.

##### 2.2.3.6. Factor analysis method.

Factor analysis refers to the study of statistical techniques for extracting common factors from groups of variables. Factors with an eigenvalue > 1 were selected. The factor loading coefficients in the factor loading matrix were differentiated towards 0 and 1 after orthogonal variance maximum rotation. Values close to 0 were ignored, and load factors greater than 0.4 were retained. We then deleted any criterion that satisfied one of the following conditions: small load factor (<0.4) on each factor; load of more than 0.4 on factors other than that to which it belonged.^[6–10]^

#### 2.2.4. Reliability and validity analysis of the PI scale.

##### 2.2.4.1. Reliability analysis of the PI scale.

###### 2.2.4.1.1. Cronbach’s α coefficient.

The use of Cronbach’s α coefficient method was the same here as during criterion screening. Cronbach’s α > 0.6 indicated good internal consistency among criteria.

###### 2.2.4.1.2. Test-retest reliability.

This method, also known as the stability coefficient, is used to indicate whether the results have changed after 2 tests to reflect the stability of the test results. As in criterion screening, a test-retest reliability result > 0.7 indicated good reliability.

##### 2.2.4.2. Validity analysis of the PI scale.

###### 2.2.4.2.1. Validity analysis.

Content validity of the scale was tested by Spearman’s rank correlation coefficient between each criterion and its own category. It is generally believed that if the correlation coefficient between each criterion and its own category is greater than that between it and other domains, the content validity of the response scale is good, and vice versa. Factor analysis was used to verify structural validity. Generally speaking, if the common factors of the scale could explain more than 50% of the variables, it was considered that the scale had good structural validity.^[11]^

#### 2.2.5. Feasibility of the PI scale.

The completion time of the scale was taken as the measurement index. It was intended that the PI scale take no more than 20 minutes to complete.^[12]^

#### 2.2.6. Responsiveness of the PI scale.

Reactivity refers to the ability of a scale to detect minor changes in quality of life,^[13,14]^ as determined by paired t test.

#### 2.2.7. Case exclusion criteria.

Those who had been confirmed to have rheumatic heart disease, congenital heart disease, cardiomyopathy, or other heart diseases after examinations;

Patients with cardiac function grade IV, severe arrhythmia, or diseases of the liver, kidney, or hematopoietic or endocrine systems;

Patients with menopausal syndrome, severe neurosis, hyperthyroidism, and other serious diseases;

Pregnant or lactating women;

Those with allergic constitution or allergies to multiple drugs;

Mentally or legally disabled patients;

Patients not treated according to ethics committee guidelines, or those for whom we had incomplete data in such a manner as could compromise evaluation of efficacy or safety.

### 2.3. Clinical validation of the PI scale

#### 2.3.1. Research subjects.

Due to the fact that the initial revision and evaluation of the PI scale, as well as the clinical validation after its development were 2 different stages of this study. In the early stage, 267 patients were selected to participate in the revision and evaluation of the PI scale, and then the preliminary developed PI scale was further validated clinically. During the clinical validation stage, a total of 41 patients with HAS (CHD with neck, shoulder, back, and leg pain, or other arthralgia) in the Second Affiliated Hospital of Shandong University of TCM were selected. 1 patient was excluded due to failure to follow-up, thus, a total of 40 cases were included. Since this study mainly observed the overall changes of 40 HAS patients before and after treatment, and determined whether the initially developed PI scale could play an exact role in clinical practice. 40 HAS patients belonged to its own pre and post control, were uniformly defined as the observation group, with 17 males and 23 females, aged 45 to 75 years. Therefore, no additional control group was established. There were no significant differences in condition, disease type, course of disease, TCM syndrome differentiation, or medication among the members of the observation group (*P* > .05). This study was conducted on the basis of patients’ knowledge and consent, which was reviewed and approved by the ethics committee of our hospital.

#### 2.3.2. Research methods.

When 40 patients with HAS received PCT in outpatient clinics, the following examinations were carried out: patients filled out the PI scale and angina pectoris grading score scale before treatment. Within 12 to 24 hours and in the 4th week, the second and third inspections were carried out, respectively. In addition, routine blood, urine, liver function, renal function, and coagulation panels were run on each patient following treatment. Each patient only received 1 PCT treatment within 1 month evaluation period. During this period, patients were allowed to take conventional medicines.

#### 2.3.3. Statistical methods.

Statistical analysis was conducted with SPSS23.0 statistical software. Each measurement index in the table was represented by (). Pearson’s correlation coefficient, paired *t* test, and repeated measurement methods were used. The scale and biochemical indexes were analyzed. The results were summarized and analyzed according to the criteria of *P* < 0.05 for correlation and *P* < 0.01 for significant correlation.

## 3. Results

### 3.1. Criteria analysis and screening results of the PI scale

#### 3.1.1. Cronbach α coefficient results.

This coefficient was used to evaluate the reliability and internal consistency of the scale. First, the overall Cronbach’s α coefficient of the scale was calculated, and then the Cronbach’s α coefficient of the scale was re-calculated after removing each criterion one at a time. The criteria whose α coefficients decreased or remained unchanged after removal were retained, while those criteria whose α coefficients increased after removal were deleted (Tables 2 and 3). Because the covariance matrix of chest pain was zero or close to zero, statistics based on its inverse matrix could not be calculated. Therefore, the system automatically deleted chest pain. The overall Cronbach’s α coefficient of the scale was 0.711. Criteria with increased α coefficient after removal included chest tightness (0.729), palpitation (0.730), redness and swelling (0.717), ecchymosis (0.715), and mass (0.719), and were thus removed from the index.

**Table 2:** Overall Cronbach’s α coefficient.

Cronbach alpha	Cronbach alpha based on normalized criterion	Number of criterion
0.711	0.699	19

**Table 3:** Cronbach’s α coefficient after deletion of criteria.

Entries	Scale average after removing criterion	Scale variance after removing criterion	Correlation between the corrected item and the total criterion	Cronbach Alpha after removing criterion
Chest tightness	9.252	9.257	−0.024	0.729
Palpitation	9.252	9.272	−0.029	0.730
Pain degree	10.141	8.144	0.419	0.685
Acidity	10.658	8.072	0.370	0.690
Numbness	10.549	8.171	0.345	0.693
Swelling	10.658	7.956	0.414	0.685
Redness and Lump	10.958	9.003	0.089	0.717
Rash point	10.906	9.009	0.202	0.706
Spot	10.904	8.919	0.264	0.703
Stagnated point	11.107	9.351	0.002	0.715
Choroid point	10.913	9.011	0.201	0.706
Granular point	10.904	8.990	0.215	0.705
Nodular point	10.906	8.966	0.232	0.704
Tenderness degree	10.130	8.061	0.456	0.681
Gritty shape	10.643	7.717	0.506	0.673
Nodular shape	10.625	7.758	0.491	0.675
Ropy shape	10.621	7.679	0.523	0.671
Lumpy shape	11.063	9.291	−0.001	0.719
Hardness	10.647	7.828	0.463	0.678

For statistical purposes, mild, moderate, and severe pain were uniformly defined as “pain degree.” Slight, moderate, and obvious tenderness were uniformly defined as “tenderness degree.”

#### 3.1.2. Test-retest reliability method results.

Each patient was measured twice, once before treatment, and once within 12 to 24 hours after. The correlation between the scores from their first and second evaluations was calculated. If the correlation coefficient was higher than 0.7, the criterion was retained; otherwise, it was eliminated. Test-retest reliability for each criterion was greater than 0.7, save for chest tightness (0.112), chest pain (0.000), and palpitation (0.409) (Table 4). These 3 criteria were thus eliminated from the PI scale.

**Table 4:** Test-retest reliability results.

Criterion	Retest reliability	Criterion	Retest reliability
Chest tightness	0.112	Stagnated point	0.937^**^
Chest pain	0.000	Choroid point	0.917^**^
Palpitation	0.409^**^	Granular point	0.804^**^
Pain degree	0.854^**^	Nodular point	0.836^**^
Acidity	0.917^**^	Tenderness degree	0.736^**^
Numbness	0.858^**^	Gritty shape	0.820^**^
Swelling	0.838^**^	Nodular shape	0.750^**^
Redness and lump	0.958^**^	Ropy shape	0.769^**^
Rash point	0.937^**^	Lumpy shape	0.943^**^
Spot	0.858^**^	Hardness	0.727^**^

**represents *P* < 0.01, there are significant correlation.

#### 3.1.3. Subjective evaluation method results.

Based on the subjective evaluation of 16 medical staff members, the 2 criteria of “redness and lump” (avg. rate importance of 46.875) and “petechiae” (avg. rate importance of 46.875) were eliminated (Table 5).

**Table 5:** Results of subjective evaluation method.

Criterion	Mean value	Criterion	Mean value
Chest tightness	65.625	Stagnated point	46.875
Chest pain	81.250	Choroid point	75.000
Palpitation	59.375	Granular point	84.375
Pain degree	87.500	Nodular point	87.500
Acidity	81.250	Tenderness degree	87.500
Numbness	81.250	Gritty shape	81.250
Swelling	75.000	Nodular shape	87.500
Redness and lump	46.875	Ropy shape	87.500
Rash point	71.875	Lumpy shape	50.000
Spot	71.875	Hardness	84.375

#### 3.1.4. Discrete trend method results.

Two criteria had SD < 0.2: as shown in Table 6, chest pain (0.00) and stagnate point (0.15); these criteria were eliminated.

**Table 6:** Results of discrete trend method.

Criterion	Standard deviation	Criterion	Standard deviation
Chest tightness	0.42	Stagnated point	0.15
Chest pain	0.00	Choroid point	0.25
Palpitation	0.42	Granular point	0.25
Pain degree	0.44	Nodular point	0.25
Acidity	0.50	Tenderness degree	0.44
Numbness	0.49	Gritty shape	0.50
Swelling	0.50	Nodular shape	0.50
Redness and lump	0.40	Ropy shape	0.50
Rash point	0.25	Lumpy shape	0.29
Spot	0.25	Hardness	0.50

#### 3.1.5. Correlation coefficient method results.

Criteria eliminated using the results of Spearman’s rank correlation coefficient analysis were: chest tightness (0.106), chest pain (0.000), palpitation (0.088), stagnated point (0.041), choroid point (0.254), granular point (0.270), nodular point (0.289), redness and lump (0.210), rash point (0.263), spot (0.322), and lumpy shape (0.069) (Table 7). Likewise, according to the results of the independent correlation coefficient, the criteria of chest pain (0.000), stagnated point (0.257), and lumpy shape (0.128) were also eliminated (Table 8).

**Table 7:** Correlation coefficient between criteria and total score.

Criterion	Correlation coefficient with total score	Criterion	Correlation coefficient with total score
Chest tightness	0.106	Stagnated point	0.041
Chest pain	0.000	Choroid point	0.254^**^
Palpitation	0.088	Granular point	0.270^**^
Pain degree	0.537^**^	Nodular point	0.289^**^
Acidity	0.500^**^	Tenderness degree	0.583^**^
Numbness	0.479^**^	Gritty shape	0.633^**^
Swelling	0.545^**^	Nodular shape	0.615^**^
Redness and lump	0.210^**^	Ropy shape	0.641^**^
Rash point	0.263^**^	Lumpy shape	0.069
Spot	0.322^**^	Hardness	0.600^**^

**represents *P* < 0.01, there are significant correlation.

**Table 8:** Correlation of criteria with total scores of other categories.

Criterion	Category 1	Category 2	Category 3	Category 4
Chest tightness	0.689^**^	0.039	0.006	−0.056
Chest pain	0.000	0.000	0.000	0.000
Palpitation	0.689^**^	−0.053	0.025	0.005
Pain degree	−0.048	0.878^**^	0.015	0.079
Acidity	−0.025	0.803^**^	0.051	0.029
Numbness	−0.004	0.707^**^	−0.015	0.135
Swelling	0.050	0.837^**^	−0.007	0.080
Redness and lump	−0.025	0.434^**^	−0.041	−0.001
Rash point	0.052	−0.001	0.838^**^	−0.056
Spot	0.029	0.003	0.853^**^	0.018
Stagnated point	0.067	−0.013	0.257^**^	−0.047
Choroid point	0.018	−0.048	0.839^**^	−0.020
Granular point	−0.020	0.030	0.850^**^	−0.057
Nodular point	0.002	0.016	0.849^**^	−0.024
Tenderness degree	−0.049	0.021	−0.005	0.853^**^
Gritty shape	−0.040	0.066	−0.011	0.835^**^
Nodular shape	−0.078	0.106	−0.077	0.819^**^
Ropy shape	−0.051	0.091	−0.009	0.835^**^
Lumpy shape	0.062	−0.009	0.102	0.128*
Hardness	−0.018	0.118	−0.114	0.819^**^

*represents *P* < 0.05, there are correlation.

**represents *P* < 0.01, there are significant correlation.

#### 3.1.6. Factor analysis results.

Since chest pain is virtually ubiquitous in patients with CHD, the variance for chest pain was 0, and it was therefore decided that “chest pain” should be removed from the index. Exploratory factor analysis was used to analyze other criteria, with factors with an eigenvalue > 1 being selected. Kaiser–Meyer–Olkin and Bartlett sphericity tests were conducted, with their results being 0.844 and 2502.3 (*P* < .01), respectively. Ultimately, our analysis yielded 5 common factors: factor 1 (granular point, choroid point, spot, rash point, nodular point), factor 2 (gritty shape, nodular shape, ropey shape, tenderness degree, hardness), factor 3 (pain degree, swelling, acidity, numbness), factor 4 (palpitation, stagnated point, redness and lump), and factor 5 (chest tightness, lumpy shape) (Table 9). After orthogonal maximum variance rotation, the cumulative variance contribution rate was 66.605%. Since the score of “redness and swelling” was -0.481 (less than our cutoff of 0.4) this criterion was deleted.

**Table 9:** Factor analysis results after maximum rotation (loading is taken as 0.4).

Criterion	Factor				
	1	2	3	4	5
Granular point	0.882				
Choroid point	0.878				
Spot	0.878				
Rash point	0.872				
Nodular point	0.864				
Gritty shape		0.875			
Nodular shape		0.870			
Ropy shape		0.864			
Tenderness degree		0.851			
Hardness		0.823			
Pain degree			0.889		
Swelling			0.875		
Acidity			0.851		
Numbness			0.676		
Palpitation				0.658	
Stagnated point				0.644	
Redness and Lump				−0.481	
Chest tightness					0.713
Lumpy shape					0.702

#### 3.1.7. Comprehensive screening results.

Based on the above 6 screening methods, we systematically eliminated corresponding criteria from the PI scale. In Table 10, selected criteria are denoted by a 1, while eliminated criteria are denoted by a 0. Criteria with a score > 4 were selected for final retention. A total of 6 criteria were eliminated. Among these was the category of CHD, along with the evaluation results of redness and lump, stagnated point, and lumpy shape. Preliminary results of the PI scale formulation are shown in Table 11.

**Table 10:** Comprehensive screening results.

Criterion	Cronbach’s α coefficient method	Test-retest reliability	Subjective evaluation method	Discrete trend method	Correlation coefficient analysis method	Factor analysis method	Total score
Chest tightness	0	0	1	1	0	1	3
Chest pain	0	0	1	0	0	1	2
Palpitation	0	0	1	1	0	1	3
Pain degree	1	1	1	1	1	1	6
Acidity	1	1	1	1	1	1	6
Numbness	1	1	1	1	1	1	6
Swelling	1	1	1	1	1	1	6
Redness and lump	0	1	0	1	0	0	2
Rash point	1	1	1	1	0	1	5
Spot	1	1	1	1	0	1	5
Stagnated point	0	1	0	0	0	1	2
Choroid point	1	1	1	1	0	1	5
Granular point	1	1	1	1	0	1	5
Nodular point	1	1	1	1	0	1	5
Tenderness degree	1	1	1	1	1	1	6
Gritty shape	1	1	1	1	1	1	6
Nodular shape	1	1	1	1	1	1	6
Ropy shape	1	1	1	1	1	1	6
Lumpy shape	0	1	1	1	0	1	4
Hardness	1	1	1	1	1	1	6

**Table 11:** The PI scale for HAS.

Pick area lookup method	Criterion	Assignment	Selection (if yes, choose √)
Patient self-examination	Acidity	1	
	Numbness	1	
	Swelling	1	
	Mild pain	0.5	
	Moderate pain	1	
	Severe pain	1.5	
Doctor’s observation	Rash point	0.5	
	Spot	0.5	
	Choroid point	0.5	
	Granular point	0.5	
	Nodular point	0.5	
Doctor’s palpation	Gritty shape	1	
	Nodular shape	1	
	Ropy shape	1	
	Hardness	1	
	Slight tenderness	0.5	
	Moderate tenderness	1	
	Obvious tenderness	1.5	
The total score			

HAS = heart arthralgia syndrome, PI = positive index.

### 3.2. Reliability and validity analysis results of the PI scale

#### 3.2.1. Reliability analysis results.

##### 3.2.1.1. Cronbach’s α coefficient method.

The overall Cronbach’s α coefficient of the scale was 0.711, indicating that the scale possessed good reliability and internal consistency (Table 2).

##### 3.2.1.2. Test-retest reliability method.

A test-retest reliability result > 0.7 means good reliability. The test-retest reliability of the 2 measurements was 0.897 (Table 12).

**Table 12:** Total test-retest reliability.

Criterion	Retest reliability
Total score 1	0.897**
Total score 2	

**represents *P* < 0.01, there are significant correlation.

#### 3.2.2. Validity analysis results.

##### 3.2.2.1. Content validity results.

Spearman’s rank correlation analysis was performed on the scores of each criterion and its respective category. There was no correlation between the criterion of chest pain and CHD (Table 13). Patient self-examination and doctor’s observation, however, were significantly correlated with the criteria belonging to them (Tables 14 and 15, *P* < .01). There was a significant correlation between doctor’s palpation and its criteria, except for lumpy shape, which had a correlation coefficient of 0.128 (Table 16, *P* < .05). The results of this analysis showed that most criteria corresponded well with the categories to which they belonged and that, overall, the scale possessed good content validity.

**Table 13:** Correlation analysis between criterion and CHD symptoms.

Criterion	Coronary heart disease symptom
Chest tightness	0.689^**^
Chest pain	0.000
Palpitation	0.689^**^

**represents *P* < 0.01, there are significant correlation.

**Table 14:** Correlation analysis between criterion and patient self-examination.

Criterion	Patient self-examination
Pain degree	0.878^**^
Acidity	0.803^**^
Numbness	0.707^**^
Swelling	0.837^**^
Redness and lump	0.434^**^

**represents *P* < 0.01, there are significant correlation.

**Table 15:** Correlation analysis between criterion and doctor’s observation.

Criterion	Doctor’s observation
Rash point	0.838^**^
Spot	0.853^**^
Stagnated point	0.257^**^
Choroid point	0.839^**^
Granular point	0.850^**^
Nodular point	0.849^**^

**represents *P* < 0.01, there are significant correlation.

**Tables 16:** Correlation analysis between criterion and doctor’s palpation.

Criterion	Doctor’s palpation
Tenderness degree	0.853^**^
Gritty shape	0.835^**^
Nodular shape	0.819^**^
Ropy shape	0.835^**^
Lumpy shape	0.128*
Hardness	0.819^**^

*represents *P* < 0.05, there are correlation.

**represents *P* < 0.01, there are significant correlation.

##### 3.2.2.2. Construct validity results.

**Figure 2.**
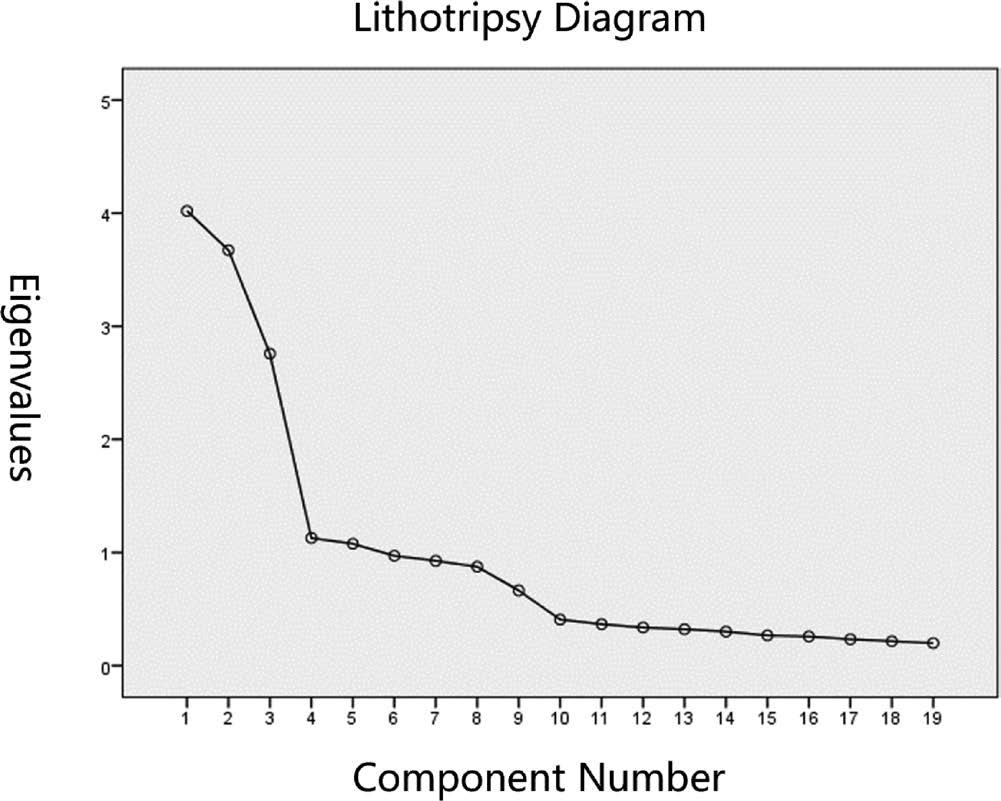
Results of common factor lithotripsy diagram. Component number is common factor; factors with eigenvalue > 1 are selected as common factors.

Based on the results of our factor analysis in Figure 2, 5 common factors were selected. Factor 1 mainly consisted of criteria related to doctor’s observation. Factor 2 mainly consisted of criteria related to doctor’s palpation. Factor 3 primarily consisted of criteria related to patient self-examination. Factor 4 was comprised of the criteria of “palpitation” and “stagnated point,” and Factor 5 consisted of “chest tightness” and “lumpy shape.” These results were slightly different from the expected structure of the scale and our original 4 categories, but they were, nevertheless, consistent with the scale overall. These 5 factors were able to explain 64.019% of the variables, indicating that the structural validity of the scale was good.

### 3.3. Feasibility analysis results

The average completion time of the PI scale, including both the patient’s self-evaluation and the physician’s examination, was 7.47 ± 3.59 minutes, making it highly feasible and easy to apply in clinical practice.

### 3.4. Reflectivity analysis results

92 patients were used not only for test-retest reliability of the PI scale, but also for the reflectivity analysis of the PI scale. Patients were reevaluated on the scale 12 to 24 hours following treatment, and the scores of the 2 tests were compared. Since the total score before and after treatment and each criterion’s score were in line with a normal distribution, the reflectivity analysis of the PI scale was analyzed by paired *t* test (Tables 17 and 18). The total score of patients after treatment was lower than before treatment. Save for the criterion of chest pain, the mean values of the other criteria had all decreased to varying degrees after treatment, but the variance did not change significantly, indicating that the dispersion degree of the 2 measurements did not change much. This indicated that the scale was relatively sensitive and had a good responsiveness degree and furthermore, that treatment of HAS with PCT can be effective in a short time.

**Table 17:** Comparison of mean values of total scores before and after treatment.

Criterion	Number of patients	Mean value	Standard deviation	*t* value	*P* value
Total score 1	92	13.00	3.15	9.224	.000
Total score 2	92	11.70	2.64		

**Table 18:** Mean values and standard deviation of each criterion before and after treatment (*x̅* ± s).

Criterion	Mean value	Standard deviation	Criterion	Mean value	Standard deviation
Chest tightness1	1.89	0.46	Chest tightness2	1.85	0.53
Chest pain1	2.00	0.00	Chest pain2	2.00	0.00
Palpitation1	1.93	0.36	Palpitation2	1.85	0.53
Pain degree1	1.00	0.46	Pain degree2	0.90	0.44
Acidity1	0.54	0.50	Acidity2	0.50	0.50
Numbness1	0.51	0.50	Numbness2	0.43	0.50
Swelling1	0.49	0.50	Swelling2	0.40	0.49
Redness and lump1	0.15	0.36	Redness and lump2	0.14	0.35
Rash point1	0.25	0.25	Rash point2	0.23	0.25
Spot1	0.25	0.25	Spot2	0.21	0.25
Stagnated point1	0.49	0.15	Stagnated point2	0.43	0.14
Choroid point1	0.25	0.25	Choroid point2	0.23	0.25
Granular point1	0.27	0.25	Granular point2	0.22	0.25
Nodular point1	0.23	0.25	Nodular point2	0.19	0.24
Tenderness degree1	1.02	0.41	Tenderness degree2	0.88	0.39
Gritty shape1	0.50	0.50	Gritty shape2	0.40	0.49
Nodular shape1	0.52	0.50	Nodular shape2	0.38	0.49
Ropy shape1	0.55	0.50	Ropy shape2	0.42	0.50
Lumpy shape1	0.11	0.31	Lumpy shape2	0.10	0.30
Hardness1	0.48	0.50	Hardness2	0.33	0.47

The first measurement denoted by a 1 following each criterion, and the second measurement is denoted by a 2 following each criterion (e.g., chest tightness 1, chest tightness 2).

### 3.5. Clinical validation results of the PI scale

#### 3.5.1. Correlation analysis results between PI scale and physical and chemical examination indexes.

Pearson’s rank correlation coefficient was used to analyze the PI scale and angina pectoris grading score scale for the first measurement. There was significant correlation between the PI score (6.18 ± 1.98) at the first measurement and angina pectoris grading score score (13.15 ± 4.25): *r* = 0.571, *P* = .000 (Table 19).

**Table 19:** Correlation between positive index scale and angina pectoris grading score scale.

Criterion	Pearson correlation coefficient	*P* value
The PI scale	0.571^**^	.000
Angina pectoris grading score scale		

PI = positive index.

**represents *P* < 0.01, there are significant correlation.

Pearson’s rank correlation coefficient method was used to analyze the PI score, angina pectoris grading score, routine blood, urine, liver function, kidney function, and coagulation panels, and other indicators measured at the second time (within 12–24 hours after treatment). There was a marginal correlation between the PI score (2.08 ± 1.52) and angina pectoris rating scale (7.35 ± 3.34): *r* = 0.288, *P* = .071. The PI scale (2.08 ± 1.52) was also correlated with thrombin time (20.95 ± 1.31): *r* = 0.343, *P* = .03 (Table 20).

**Table 20:** Correlation results between second measurement of the positive index scale and each index.

Index	Correlation coefficient with the PI scale	*P* value	Index	Correlation coefficient with the PI scale	*P* value
2 Angina pectoris rating score scale	0.288	.071	2 Red blood cell count	0.145	.373
2 White blood cells	0.079	.628	2 Hemoglobin	0.183	.258
2 Bilirubin	0.000	.000	2 Platelet count	−0.009	.955
2 Glucose	0.000	.000	2 Alanine aminotransferase	−0.047	.772
2 Occult blood	−0.088	.589	2 Aspartate aminotransferase	−0.176	.277
2 Total white blood cell count	0.032	.844	2 Total bilirubin	0.238	.139
2 Lymphocyte ratio	0.103	.527	2 Glutamyl transpeptidase	0.191	.237
2 Neutrophils	−0.035	.829	2 Alkaline phosphatase	0.083	.611
2 Lymphocytes	0.133	.414	2 Urea nitrogen	−0.197	.223
2 Monocytes	−0.002	.988	2 Creatinine	−0.044	.788
2 Eosinophils	−0.014	.932	2 Prothrombin time	0.066	.686
2 Basophils	−0.162	.317	2 Thrombin time	0.343*	.030

For the convenience of statistics in the table, 2 was added before the second measurement index (e.g., 2 Angina Pectoris Grading Scale).

PI = positive index.

*represents *P* < 0.05, there are correlation.

Once again, Pearson’s rank correlation coefficient method was used to analyze the PI scale, angina pectoris grading score scale, routine blood, urine, liver function, kidney function, and coagulation panels a third time, 4 weeks after treatment. This time there was no correlation between the PI score (0.51 ± 0.50) and the angina pectoris grading score (4.20 ± 3.38): *r* = 0.165, *P* = .308 (Table 20). There was, however, correlation between the PI score and alanine aminotransferase (*r* = 0.374, *P* = .017 < 0.05) and lymphocytes (*r* = 0.323, *P* = .042). The PI score was also negatively correlated with thrombin time (*r* = −0.360, *P* = .023). Further correlations among the dimensions of routine blood, urine, liver function, kidney function, and coagulation panels were found (Table 20 and 21), but these were of low analytical value, and will not be detailed.

**Table 21:** Correlation results between third measurement of positive index scale and each index.

Index	Correlation coefficient with the PI scale	*P* value	Index	Correlation coefficient with the PI scale	*P* value
3 Angina pectoris rating score scale	0.165	0.308	3 Red blood cell count	0.039	0.813
3 White blood cells	−0.166	0.305	3 Hemoglobin	0.230	0.154
3 Bilirubin	0.000	0.000	3 Platelet count	−0.031	0.848
3 Glucose	−0.215	0.183	3 Alanine aminotransferase	0.374*	0.017
3 Occult blood	−0.093	0.569	3 Aspartate aminotransferase	0.210	0.193
3 Total white blood cell count	−0.099	0.544	3 Total bilirubin	0.207	0.201
3 Lymphocyte ratio	0.300	0.060	3 Glutamyl transpeptidase	0.148	0.363
3 Neutrophils	−0.176	0.279	3 Alkaline phosphatase	0.122	0.455
3 Lymphocytes	0.323*	0.042	3 Urea nitrogen	−0.058	0.723
3 Monocytes	−0.130	0.425	3 Creatinine	−0.153	0.345
3 Eosinophils	0.095	0.558	3 Prothrombin time	−0.164	0.313
3 Basophils	−0.087	0.593	3 Thrombin time	−0.360*	0.023

For the convenience of statistics in the table, 3 was added before the third measurement index (e.g., 3 Angina Pectoris Grading Scale).

PI = positive index.

*represents *P* < 0.05, there are correlation.

#### 3.5.2. Comparative analysis results of each index before and after measurement.

The 3 measurement results of the PI scale and angina pectoris grading scale were compared. The average score and SD of the PI scale gradually decreased over each measurement (6.18 ± 1.98, 2.08 ± 1.52, and 0.51 ± 0.50, respectively; *F* = 225.57, *P* = .000) (Table 22). The angina pectoris grading score also decreased over the 3 measurements (13.15 ± 4.25, 7.35 ± 3.34, and 4.20 ± 3.38; *F* = 175.16, *P* = .000) (Table 23). The 3 measurement values of the 2 tables were each statistically significantly different, thus demonstrating that PCT can effectively relieve the symptoms of CHD while treating arthralgia syndrome. Thus, PCT is effective in the treatment of HAS.

**Table 22:** Results of 3 consecutive measurements of the positive index scale.

Index	Mean value	Standard deviation	*F* value	*P* value
The PI scale	6.18	1.98	225.57	.000
2 The PI scale	2.08	1.52		
3 The PI scale	0.51	0.50		

To distinguish between the second and third consecutive measurements, 2 and 3 are added before the second and third measurement indicators (i.e., 2 The PI scale, 3 The PI scale).

PI = positive index.

**Table 23:** Results of 3 consecutive measurements of the angina pectoris grading score scale.

Index	Mean value	Standard deviation	*F* value	*P* value
The angina pectoris grading score scale	13.15	4.25	175.16	.000
2 The angina pectoris grading score scale	7.35	3.34		
3 The angina pectoris grading score scale	4.20	3.38		

To distinguish between the second and third consecutive measurements, 2 and 3 are added before the second and third measurement indicators (i.e., 2 the angina pectoris grading score, 3 the angina pectoris grading score).

Paired sample *t* test was used to compare the second and third physical and chemical examinations. The results showed that there were no significant changes in routine blood, urine, liver function, kidney function, or coagulation panels, indicating that the negligible effect of PCT on the liver, kidney, and other viscera of patients with HAS. These results indicate that PCT is safe and can be widely used in clinical practice (Table 24).

**Table 24:** Comparison of second and third routine blood, urine, liver function, kidney function, and coagulation panels.

Index	*t* value	*P* value	Index	*t* value	*P* value
Angina pectoris rating score scale	1.583	.112	Hemoglobin	−0.319	.752
White blood cells	0.000	.000	Platelet count	1.408	.167
Bilirubin	−1.292	.204	Alanine aminotransferase	−0.808	.424
Glucose	0.243	.809	Aspartate aminotransferase	−0.054	.957
Occult blood	0.491	.626	Total bilirubin	−1.177	.247
Total white blood cell count	0.598	.553	Glutamyl transpeptidase	−1.897	.065
Lymphocyte ratio	−0.048	.962	Alkaline phosphatase	−0.882	.383
Neutrophils	−0.981	.333	Urea nitrogen	−1.500	.142
Lymphocytes	−1.032	.308	Creatinine	−1.672	.102
Monocytes	1.346	.186	Prothrombin time	1.501	.141
Eosinophils	1.076	.289	Thrombin time	0.781	.439
Basophils	−0.239	.812			

## 4. Discussion

A number of different scales for measuring CHD and its associated symptoms have been developed by Western and TCM, many of which are widely used in clinical practice. Some studies have suggested that the scoring results of remote care and its related scales were related to the incidence of cardiovascular events, CHD, and hypertension. However, many of these studies lacked final clinical validation.^[15–18]^ When developing the PI scale, we drew from different research methods such as the formulation, screening and assignment of scales in Western medicine and TCM.

While these scales can evaluate the efficacy and prognosis of coronary heart disease (CHD), few studies have developed a scale suitable for CHD patients with arthralgic symptoms such as neck, shoulder, waist, and leg pain. Therefore, we sought to develop the PI scale to fill this gap, one in line with China’s national conditions on a comprehensively representative operational scale.

In this study, 6 different criterion screening methods were used, and 18 criteria were finally obtained. Finally, the preliminary formulation of the PI scale was completed. For the 3 criteria of chest tightness, chest pain, and palpitation, the Cronbach’s α coefficient, test-retest reliability, and correlation coefficient methods suggested that they should be deleted. As can be seen in Tables 12 and 13, the score for “redness and lump” and “stagnated point” was only 2 points, indicating that they should be removed from patient self-examination and the doctor’s observation, respectively. In practice, some patients have difficulty distinguishing between the criteria of “swelling” and “lump” in dimensions. Similarly, doctors in our study reported that the categories of “stagnated point” and “choroid point” were slightly redundant. Moreover, doctors said that the utility of the criterion “lumpy shape” was relatively poor, as there were few lumps in the clinic, and those that did appear were mostly in the severe stage of the disease. Consequently, “redness and lump,” “stagnated point,” and “lumpy shape” were removed from the index.

The scale was divided into 5 common factors by factor analysis. These results were slightly different from the expected structure of the scale and our original 4 categories. Doctors believed that “palpitation” and “chest tightness” belonged to the category of CHD. The correlation between “palpitation” and “stagnated point” was small, as “stagnated point” more closely corresponds to the contents of the doctor’s observation. Likewise, “chest tightness” was less related to “lumpy shape,” which was more inclined to the contents of doctor’s palpation. After comprehensive consideration of various analysis methods, we deleted the criteria of “chest tightness,” “chest pain,” “palpitation,” “redness and lump,” “stagnated point,” and “lumpy shape.” The final scale was divided into 3 categories: patient self-inspection, containing the criteria of acidity, numbness, swelling, mild, moderate, and severe pain; doctor’s observation, consisting of the criteria of rash point, spot, choroid point, granular point, and nodular point; and doctor’s palpation, which contained the criteria of gritty shape, nodular shape, ropy shape, hardness, and slight, moderate, and obvious tenderness.

Four methods, consisting of the Cronbach’s α coefficient, test-retest reliability, Spearman’s rank correlation coefficient, and exploratory factor analysis methods, showed that the PI scale possessed good reliability and validity. Moreover, for the 92 patients who were repeatedly measured on the PI scale within 12 to 24 hours, the degree by which each criterion was lower differed, indicating that the scale was highly sensitive, well reflected, and could accurately measure differences. It can also be explained that PCT can be effective in treating patients with HAS in a short period of time.

In the clinical validation phase, we included 40 outpatient clinical HAS patients to further verify whether the preliminarily developed PI scale could guide clinical practice. Clinical validation results of the scale showed that the total score of patients within 12 to 24 hours after treatment with PCT was lower than that of patients before treatment, and that the average value of each criterion after treatment was lower than that before treatment (*P* < .01). That the scale was able to detect changes within 12 to 24 hours of treatment shows that the sensitivity and response of the scale are good and that PCT can, in a short time, have obvious effects on the symptoms of HAS. As time goes on, the PI score gradually decreased, indicating that PCT has a good effect on long-term patient prognosis.

In the clinical validation phase, compared with the angina pectoris grading scale score, the pre- and post-changes of the PI scale initially developed in the 3 measurements were more obvious. The first measurement showed a significant correlation between the 2 scales, whereas the second and third measurements did not. This is likely due to the fact that, while angina pectoris is a concomitant symptom to arthralgia syndrome, it has a relatively long remission time. Therefore, the correlation between the 2 scales gradually decreases over time following PCT treatment.

In the clinical validation phase, comparison of the second and third physical and chemical examinations showed that there was no significant change in the results of routine blood, urine, liver function, kidney function, or coagulation panels. This result demonstrates that the treatment had no detectable impact on the functions of the liver, kidney, and other viscera. The results of the last 2 measurements showed that there was a significant correlation between the PI scale and thrombin time. This may be related to the need to pick out separate Yang collaterals and extrude the blood or tissue fluid, which could change coagulation time accordingly.

In this discussion, we are more inclined to explain and discuss the relevant research results of the PI scale. As there is no corresponding scale for HAS patients in the current research scale, many published articles on the scale development of other diseases have relatively one-sided references for the preliminary developed PI scale, so there are relatively few citations.

The age range of patients throughout this study was between 15 and 85 years, indicating that using PCT to treat HAS can benefit patients in a highly broad age range. The short- and long-term efficacy of PCT for treating HAS was significant. The PI scale, in its current, preliminary form, can be widely used to evaluate the efficacy of PCT in the clinic.

## 5. Conclusion

We initially developed and applied a clinically validated the PI scale in this study. This scale had good reliability, validity, responsiveness, and strong clinical operability. This scale plays an important role in guiding the implementation of PCT, which can be widely used as a tool to evaluate the initial efficacy of PCT. The sample size used in our study was relatively small; the final scale will need to be evaluated in a larger sample size. Ideally, this will occur in a multicenter clinical trial to better enhance the relevance of the scale to the general HAS patient population.

## Acknowledgments

Thanks to the book: the Advanced Course of SPSS Statistical Analysis.

## Author contributions

**Investigation:** Sai Xu.

**Validation:** Shouqiang Chen.

**Writing – original draft:** Sai Xu, Shouqiang Chen.

**Writing – review & editing:** Yunsheng Xu.
